# Targeting the host response in sepsis: current approaches and future evidence

**DOI:** 10.1186/s13054-023-04762-6

**Published:** 2023-12-06

**Authors:** Christian Bode, Sebastian Weis, Andrea Sauer, Pedro Wendel-Garcia, Sascha David

**Affiliations:** 1 Department of Anesthesiology and Intensive Care Medicine, University Hospital Bonn, Venusberg-Campus 1, 53127 Bonn, Germany; 2 Institute for Infectious Disease and Infection Control, University Hospital Jena, Friedrich-Schiller University Jena, Jena, Germany; 3 Department of Anesthesiology and Intensive Care Medicine, University Hospital Jena, Friedrich-Schiller University Jena, Jena, Germany; 4 Leibniz Institute for Natural Product Research and Infection Biology, Hans-Knöll Institute-HKI, Jena, Germany; 5 Institute of Intensive Care Medicine, University Hospital Zurich, Zurich, Switzerland

**Keywords:** Septic shock, Clinical studies, Disease tolerance, Immunomodulation, Immunotherapy, Biomarkers, Precision medicine, Immunosuppression, Personalized medicine

## Abstract

Sepsis, a dysregulated host response to infection characterized by organ failure, is one of the leading causes of death worldwide. Disbalances of the immune response play an important role in its pathophysiology. Patients may develop simultaneously or concomitantly states of systemic or local hyperinflammation and immunosuppression. Although a variety of effective immunomodulatory treatments are generally available, attempts to inhibit or stimulate the immune system in sepsis have failed so far to improve patients’ outcome. The underlying reason is likely multifaceted including failure to identify responders to a specific immune intervention and the complex pathophysiology of organ dysfunction that is not exclusively caused by immunopathology but also includes dysfunction of the coagulation system, parenchymal organs, and the endothelium. Increasing evidence suggests that stratification of the heterogeneous population of septic patients with consideration of their host response might led to treatments that are more effective. The purpose of this review is to provide an overview of current studies aimed at optimizing the many facets of host response and to discuss future perspectives for precision medicine approaches in sepsis.

## Introduction

**Fig. 1:**
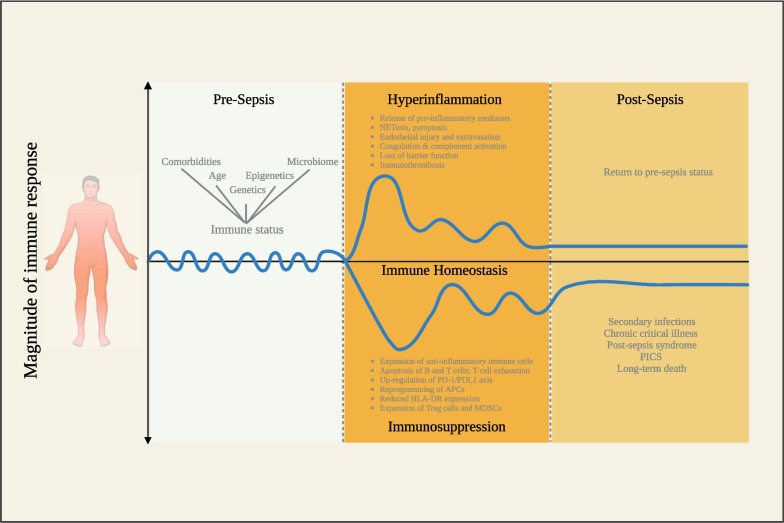
Model of sepsis-induced immune responses. This extended model of sepsis-induced immune responses describes the host inflammatory response before, during, and after sepsis. Infection modifies the innate and adaptive immune response for sustained periods of time, even long after clinical recovery. The immune response in sepsis is highly personalized and contingent upon the patient's immune status when infection occurs. This status is influenced by various factors including age, comorbidities, environmental elements, and the microbiome. Moreover, each patient exhibits a highly intricate combination of genetic variations and epigenetic alterations, rendering their immune system a virtually unique selection of genes responsible for cytokines and mediators that regulate immune responses. Excessive inflammation is triggered by the release of pro-inflammatory mediators by various cell types upon detecting pathogen-associated molecular patterns (PAMPs). Simultaneously, the activation of the complement system, the vascular endothelium, and the coagulation system results in microcirculatory disturbances. These processes are exacerbated by the release of damage-associated molecular patterns (DAMPs) as a consequence of tissue damage, the discharge of neutrophil extracellular traps (NETosis), and inflammatory cell death (pyroptosis). Immune suppression can develop at various time points and is characterized by the secretion of anti-inflammatory cytokines, the apoptosis of T cells, B cells, and dendritic cells, T cell exhaustion, and the proliferation of anti-inflammatory immune cells like regulatory T cells (Tregs) and myeloid-derived suppressor cells (MDSCs). Immune suppression is further intensified by decreased expression of human leukocyte antigen–antigen D related (HLA-DR) and heightened expression of programmed cell death 1 (PD-1) and its corresponding ligand (PD-L1). Post sepsis, the immune response can return to pre-sepsis status; however, many sepsis survivors later succumb to secondary infections, chronic critical illness, post-sepsis syndrome, and post-intensive care syndrome (PICS), severely impacting quality of life. A persistent sepsis-induced immune dysfunction can eventually lead to long-term death

Sepsis remains a leading cause of death worldwide, despite our advances in critical care medicine [1]. First immunotherapeutic approaches that aimed at controlling the early hyperinflammatory phase were not successful in clinical trials. Subsequent deeper insight into the pathophysiology revealed that systemic hyperinflammation, characterized by high levels of circulating pro-inflammatory markers such as cytokines or ferritin and the concomitant presence of organ dysfunction, does not necessarily characterize all sepsis patients. Instead, some patients are found to be rather systemically immunosuppressed. The common denominator infection-associated organ dysfunction can also occur independently of these two extremes and local immune responses may vary from the blood compartment (Fig. 1) [2, 3]. As a consequence, sepsis was redefined as a dysregulated host response to infection [4]. In medicine, the field of immunotherapeutics for other disease has rapidly evolved, leading to countless effective treatment strategies, *e.g*., to control tumor growth or limit autoimmunity [5]. As an analogy, the development of specific adapted therapies targeting the dysregulated host response in sepsis may improve the outcome of some of our patients. Potentially, it is the heterogeneity of the syndrome and the associated difficulties in matching the right patient to a given treatment that resulted in little success in the clinical setting so far [6]. Here, we provide an overview of current approaches to target the many facets of the host response and discuss future perspectives in the field of precision immunotherapy.

## Targeting hyperinflammation

### Selective immunomodulators

#### Tumor necrosis factor

Tumor necrosis factor (TNF) plays a crucial role in the systemic inflammatory response, and biologics that neutralize TNF are among the most successful drugs for the treatment of various chronic inflammatory diseases [7]. However, initial clinical trials targeting TNF in sepsis patients yielded disappointing results [8–12]. A meta-analysis of 17 randomized controlled trials (RCTs) involving more than 8000 septic patients treated with anti-TNF showed a small but significant reduction in 28-day all-cause mortality [13]. Interestingly, in a study of 2634 sepsis patients, treatment with the anti-TNF antibody (Ab) afelimomab resulted in a modest but significant reduction in 28-day mortality if serum IL-6 levels were > 1000 pg/mL, while patients with lower IL-6 levels did not benefit from treatment [14]. This suggests that a specific subset of patients identified by biomarkers may benefit from anti-TNF therapy.

#### Interleukin-1 receptor

IL-1 signaling is mediated by the two distinct ligands IL-1α and IL-1β, both of which act on the IL-1 receptor (IL-1R) to trigger inflammation [15]. While IL-1 β is mainly released by activated immune cells, IL-1α is a nearly ubiquitous alarmin released by injured tissue. There has long been interest in the deleterious role of IL-1R signaling in sepsis, but RCTs did not show a significant prolongation of survival [16, 17]. Interestingly, a retrospective analysis of 529 sepsis patients found that anakinra significantly reduced mortality when baseline plasma IL-1RA levels were above 2071 pg/mL [18]. In a further re-analysis of an multicenter (m)RCT, 763 patients were re-grouped according to the presence of features of macrophage activation syndrome (MAS) in the form of disseminated intravascular coagulation (DIC) and hepatobiliary dysfunction (HBD) [19]. In this study, anakinra was associated with a significant improvement in survival of patients with sepsis and concomitant HBD/DIC. Recently, the mRCT SAVE-MORE has stratified coronavirus disease 2019 (COVID-19) patients with mild to severe pneumonia according to a soluble urokinase-type plasminogen activator receptor (suPAR) level ≥ 6 ng/mL and tested anakinra compared to standard of care (SoC) [20]. Anakinra treatment provided higher odds for clinical improvement and lowered the 28-day mortality from 6.9 to 3.2%. SuPAR and MAS features illustrate the biological and clinical consequences of hyperinflammation such as coagulopathy and tissue damage [21]. The results of the above studies therefore suggest that these classes of biomarkers may enable more targeted anakinra treatment in sepsis.

#### Interleukin-6

Interleukin-6 (IL-6) is another important cytokine involved in the innate immune response in sepsis [6]. IL-6 inhibitors are the approved treatment for the hyperinflammatory state of CAR-T cell-induced cytokine release syndrome [22]. Recently, IL-6 inhibition has been studied in the COVID-19 pandemic with conflicting results. However, two studies pooling data from previous trials involving more than 10,000 critically ill COVID-19 patients show that IL-6 inhibitor administration was associated with lower 28-day all-cause mortality [23, 24]. Although it is currently unclear whether IL-6 inhibition has similar benefits in other cases of sepsis, a recent Mendelian randomization analysis suggests that IL-6 receptor blockade was associated with lower mortality in 11,643 patients of the UK Biobank cohort with non-COVID-19 sepsis [25]. Overall, these data suggest that a mRCT of IL-6 inhibition in sepsis, ideally as part of a predictive enrichment approach, should at least be considered.

#### Complement inhibition (anti-C5a)

The complement system is a key regulator of immunity that bridges the innate to the adaptive response, and that contributes to opsonization and lysis of invading pathogens. The complement cascade can by activated via three pathways by invading pathogens and also via, e.g., tissue damage and the associated release of endogenous danger molecules (DAMPs) [26]. Normally, the complement system plays a protective role but can also directly contribute to a hyperinflammatory state triggering the development of complications like multiple-organ failure. Experimental studies have linked hyperinflammation and endothelial barrier breakdown with complement activation and some trials have shown benefits using inhibitory strategies in non-human primates and pigs with regard to the incidence of organ failure, coagulopathy, and even survival [27–30]. A phase IIa mRCT (SCIENS-trial) investigated complement inhibition in sepsis using three different doses of a monoclonal anti-C5a antibody (vilobelimab). This pharmacodynamics/-kinetic study demonstrated efficient inhibition of C5a and some secondary efficacy endpoints. The authors reported that patients receiving higher dosages of vilobelimab had more intensive care unit (ICU)-, vasopressor-, and ventilator-free days [31] (Table 1).

**Table 1:** Recent clinical studies that target hyperinflammation in sepsis*

Anti-inflammatory agent	Mechanism of action	Study population	Primary outcome	Results	Author, year of publication or NCT number
Vitamin C	Inhibition of Nf-kb activation and HMGB1 release, enhancement of chemotaxis and phagocytosis	*n* = 872, proven or suspected infection (ICU stay < 24 h) and vasopressor therapy	Composite of death or persistent organ dysfunction on day 28	Higher risk of death or persistent organ dysfunction on day 28	Lamontagne et al. 2022 [42]
		*n* = 167, sepsis and ARDS (< 24 h)	Modified SOFA score at 96 h, CRP and thrombomodulin levels at 168 h	No significant difference in primary endpoints	Fowler et al. 2019 [40]
Hydrocortisone, vitamin C, thiamine	Pleiotropic immunomodulatory effects (e.g., inhibition of NF-kB, AP-1, endothelial and neutrophil activation)	*n* = 216, early septic shock (< 24 h)	Time alive and free of vasopressors on day 7	No significant difference in primary endpoints	Fujii et al. 2020 [41]
Clarithromycin	Wide-ranging immunomodulatory effects (e.g., inhibition of TLR expression and signaling, inhibition of pro-inflammatory cytokine, chemokine and DAMPs release)	*n* = 110, sepsis, ARDS and multiple-organ dysfunction	28-day mortality	No significant difference, lower incidence of sepsis recurrence, significant increase in monocyte HLA-DR expression; expansion of non-classical monocytes; and upregulation of genes involved in cholesterol homeostasis	Karakike et al. 2022 [51]
Vilobelimab	Novel monoclonal anti-C5a antibody	*n* = 72, sepsis or septic shock	pharmacodynamics, pharmacokinetics, safety	Dose-dependent neutralization of C5a, higher ICU- and ventilator-free days	Bauer et al. 2021 [31]
Thrombomodulin (ART-123)	Binding of thrombin, activation of protein C, inhibition of DAMPs associated inflammation and organ injury	*n* = 800, sepsis-associated coagulopathy	28-day mortality	No significant difference	Vincent et al. 2019 [58]
Adrecizumab (HAM8101)	Non-neutralizing adrenomedullin antibody	*n* = 301, septic shock	Safety (90-day mortality, adverse events), tolerability	No significant difference in 90-day mortality and adverse events, well tolerated	Laterre et al. 2021 [63]
Cytokine adsorption	Adsorption of PAMPs, DAMPs and cytokines	*n* = 2611, septic shock, cardiac arrest, cardiopulmonary bypass surgery, severe illness	Longest reported mortality	No significant difference	Becker et al. 2023 [67]
Coupled plasma filtration and adsorption	Adsorption of pro-inflammatory and anti-inflammatory mediators	*n* = 96, septic shock	Adsorption of IL-6, vasopressor requirements, 30-day mortality	No difference in IL-6 and vasopressor requirements, increased mortality hazard ratio	Wendel Garcia et al. 2021 [65]
		*n* = 115, septic shock	Mortality at hospital discharge	Significantly higher mortality, trial stopped for futility	Garbero et al. 2021 [66]
Therapeutic plasma exchange (TPE)	Elimination of pro-inflammatory and replacement of protective molecules	*n* = 40, early septic shock (< 24 h)	Early hemodynamic improvement	Significant hemodynamic improvement over first 24 h, higher baseline lactate levels were predictive for more efficient hemodynamic improvement; significant reduction of procalcitonin (PCT), soluble receptor of tyrosine kinase with immunoglobulin-like and EGF-like domains (sTie-2), von Willebrand factor antigen (vWF:Ag); significant repletion of antithrombin-III, ADAMTS13, protein C	Stahl et al. 2022 [68] David et al. 2021 [70]
		*n* = 20, early septic shock (< 24 h)	Levels of endothelial glycocalyx degradation products (heparan sulfate (HS), heparinase (Hpa)-1 and -2)	HS levels significantly reduced, significantly normalized Hpa-2/Hpa-1 ratio, attenuation of eGC damage ex vivo with serum from TPE group	Stahl et al. 2021 [72]
		*n* = 80, septic shock and evidence of multiple-organ failure	28-day mortality	Significantly reduced 28-day mortality	Keith et al. 2020 [69]
		*n* = 274, early septic shock (< 24 h)	28-day mortality	Not yet recruiting	NCT05726825 (EXCHANGE-2)
		*n* = 80, early septic shock (< 24 h)	Feasibility of a large, multicenter trial of TPE in patients with septic shock	Not yet recruiting	NCT05093075 (PLEXSIS)

*A summary of clinical studies over last past 5 years

### Non-selective immunomodulators

#### Corticosteroids

Glucocorticoids have potent anti-inflammatory properties such as inhibition of innate immune response and endothelial activation [32]. Clinical trials of glucocorticoids in sepsis yielded controversial results, with some showing improved outcomes and others reporting no or even adverse effects [33]. Therefore, current guidelines contain only a weak recommendation for hydrocortisone in septic shock [34]. Recently, however, a clear indication for dexamethasone in severe COVID-19 has been established, shedding new light on the efficacy of glucocorticoids in a homogenous population of critical ill patients [35–37]. In addition, recent data, including the CAPE-COD trial, showed that patients with severe community-acquired pneumonia who received hydrocortisone had a lower mortality rate [38, 39]. These promising data from specific patient populations may also lead to a renaissance of glucocorticoid therapy in the context of subgrouping sepsis patients [37].

#### Vitamin C

Vitamin C is an antioxidant with pleiotropic anti-inflammatory activity that is depleted in response to oxidative stress, which is one reason to investigate the effect of vitamin C, either alone or in random combinations with hydrocortisone and thiamine [20]. While initial studies suggested improved outcomes in sepsis [6], further studies could not confirm a beneficial effect. The CITRIS-ALI RCT showed that vitamin C did not significantly improve organ dysfunction scores or inflammatory markers in patients with sepsis and ARDS [40]. Similarly, the VITAMINS trial, which examined the use of vitamin C in septic shock, found no significant improvement in survival without vasopressor administration for 7 days [41]. The LOVIT trial, an RCT including patients with septic shock, found that vitamin C therapy increased the risk of a composite end point-death or persistent organ dysfunction at day 28 [42]. Yet, recent meta-analyses found an improvement in delta—sequential organ failure assessment (delta-SOFA) score and a reduction in the duration of vasopressor use, whereas short-term mortality was not affected [43, 44]. Because the role of vitamin C in sepsis remains uncertain, it should only be used in the context of RCTs. Such studies are underway and may provide more insight into optimal dosing and treatment duration, as well as the patient population that will benefit most from vitamin C therapy (Table 1).

#### Antibiotics with anti-inflammatory properties

In addition to their antibacterial action, tetracyclines and macrolides in particular exert pleiotropic immunomodulatory effects that may be able to limit the hyperinflammatory response in patients with sepsis. In experimental sepsis, tetracyclines limit the inflammasome-caspase-1 pathway and promote disease tolerance to infection [45–48]. In a RCT of 231 dengue fever patients, treatment with doxycycline was associated with lower mortality, which correlated positively with lower levels of pro-inflammatory cytokines [49].

Recent studies using macrolides in acute respiratory distress syndrome (ARDS) patients have shown a survival benefit and shorter time to successful discontinuation of mechanical ventilation [50]. An mRCT found no effect of clarithromycin on mortality in sepsis patients with respiratory and multiple-organ dysfunction [51] (Table 1). However, clarithromycin was associated with a lower recurrence of sepsis, a significant increase in monocyte human leukocyte antigen-DR isotype (HLA-DR) expression, and an expansion of monocytes, suggesting a possible role for macrolides in immune recovery [51].

#### (Activated) protein C and thrombomodulin

A controlled interaction between the endothelium, the immune, and the coagulation system is a conserved and physiologically required process. Nevertheless, in dysregulated settings it can spark systemic microvascular clotting, often referred to as immunothrombosis [52]. In sepsis, these phenomena can contribute to disseminated intravascular coagulation (DIC), thus further damaging tissues and organs opening potential avenues for therapeutic targets [6]. Yet, anticoagulants like heparin and P2Y12 inhibitors show only variable benefits accompanied by high bleeding risks [53].

Activated protein C (APC) is a naturally occurring anticoagulant that when given as the recombinant form (Xigris [drotrecogin alfa]) inhibits and reduces the expression of tissue factor; it was the first biologic specific agent to be approved for the treatment of severe sepsis and septic shock based on the PROWESS trial that showed a reduction in 28-day mortality [54]. These results could not be replicated in subsequent trials, ultimately leading to the withdrawal of APC in 2011 from the market [55, 56].

More recently, the focus has been on recombinant thrombomodulin (ART-123), which promotes protein C activation and has additional anti-inflammatory properties [57]. However, treatment with ART-123 did not improve survival in three RCTs [58–60]. A recent meta-analysis of these trials found that ART-123 reduced 28-day mortality only in a subgroup of patients with evidence of sepsis-associated coagulopathy [61].

#### Bioactive adrenomedullin

**Fig. 2:**
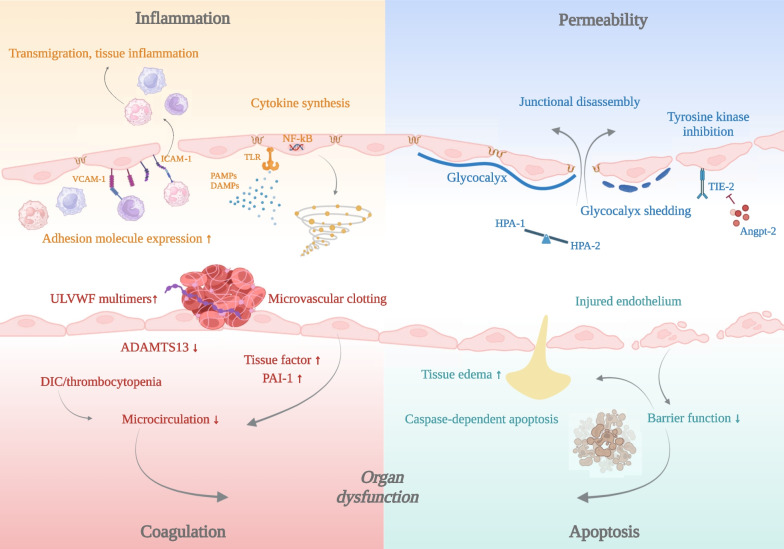
Vascular endothelial dysfunction in the pathogenesis of septic organ injury. The vascular endothelium plays a crucial role in inflammation, immunothrombosis, and vascular barrier integrity. During sepsis, the activation of a highly complex inflammatory cascade by pathogen-associated molecular patterns (PAMPs) and damage-associated molecular patterns (DAMPs) triggers the production of pro-inflammatory, proapoptotic, and procoagulant mediators by both immune cells and vascular endothelial cells (ECs). Toll-like receptor (TLR) signaling causes nuclear translocation of transcription factor NF-kb, leading to a deleterious cytokine release syndrome. The luminal surface of the vascular endothelium is lined by the endothelial glycocalyx (eGC), a gel-like carbohydrate-rich structure. In sepsis, heparanase-1 (HPA-1) activity is upregulated inducing degradation of the eGC. Glycocalyx shedding exposes embedded adhesion molecules such as intracellular adhesion molecule-1 (ICAM-1) and vascular adhesion molecule-1 (VCAM-1) which both enable leukocyte rolling, adhesion, and transmigration. Loss of the eGC, junctional disassembly, and EC apoptosis result in capillary barrier dysfunction, increased permeability, and interstitial tissue edema. Besides amplifying the inflammatory host response, ECs also promote a prothrombotic state leading to microvascular clotting and frequently disseminated intravascular coagulation (DIC). A lack of cleavage of von Willebrand factor (VWF) due to reduced levels of a disintegrin and metalloproteinase with a thrombospondin type 1 motif, member 13 (ADAMTS13) contributes to the accumulation of ultra-large VWF (ULVWF) multimers facilitating platelet adhesion to injured endothelium. The upregulation of tissue factor which initiates extrinsic coagulation and plasminogen activator inhibitor 1 (PAI-1), the main inhibitor of fibrinolysis, further augments the process of sepsis-induced immunothrombosis

The response of the endothelium to inflammatory stimuli is per se an evolutionary-derived protective mechanism to control infections. Any of its physiological function can be affected [62]. As a net result, the quiescent “healthy” endothelium changes toward a procoagulant, pro-adhesive, pro-inflammatory, and hyper-permeable phenotype together with a macrovascular vasoplegia (Fig. 2). All these alterations are part of a complex physiological response to an infection, but the simultaneous and systemic occurrence can have fatal consequences.

Among all endothelial alterations, systemic capillary leakage is a particularly relevant player in the pathophysiology of septic multiple-organ failure. The molecular mechanisms involved in the formation of gaps between adjacent endothelial cells (EC) are tightly controlled by a variety of proteins that might serve as therapeutic targets. As an example, bioactive adrenomedullin (bioADM) is a small molecule with differential functions dependent on both its localization (intra-/ or extravascular) and the target cell (EC or vascular smooth muscle cells (VSMC)). Intravascular bioADM has protective anti-permeability effects. However, if it is localized outside the vasculature this protection is lost triggering increased permeability. Simultaneously, extravascular bioADM promotes VSMC relaxation thereby aggravating hypotension and shock. Adrecizumab is a non-functional antibody to bioADM. Ligation increases its size thereby losing the capability to migrate to the interstitial space but maintaining its beneficial barrier protective effects. A recent feasibility RCT (AdrenOSS-2) confirmed not only safety of the substance but also some promising signals with regard to secondary efficacy endpoints [63, 64] (Table 1).

#### Extracorporeal blood purification

The field of extracorporeal strategies to modulate the host response has been growing over the last decade. Focusing on adsorptive technologies, it has been postulated that the removal of pro-inflammatory mediators during early and severe septic shock might be beneficial. Numerous uncontrolled reports support this notion, but evidence from controlled trial is sparse. Besides a few negative trials and trials that even indicated potential harm [65, 66], a 2023 meta-analysis including RCTs and propensity matched analysis did not show any benefit but also no harm with regard to mortality [67]. Therapeutic plasma exchange (TPE) has shown some promising results in removing injurious and replacing protective but consumed proteins thereby rebalancing hemostasis in septic shock [68–70]. This approach does not only address the immune response but also targets the well establish link to coagulopathy and vascular barrier breakdown. Two examples are the von Willebrand (VWF) system and the endothelial glycocalyx (eGc).

First, to avoid microangiopathic obstruction of the microcirculation, systemically released VWF is enzymatically cleaved by a disintegrin and metalloproteinase with a thrombospondin type 1 motif, member 13 (ADAMTS13). During septic shock, this process consumes ADAMTS13 aggravating microvascular clotting and consequently organ malperfusion. Second, the eGC is a gel-like layer that mostly consists of sugars such as proteoglycans and glycosaminoglycans that regulates inflammation, permeability, and coagulation. In sepsis, a distinct regulation of counteracting enzymes (i.e., heparanases) can lead to massive degradation of the eGC [71].

TPE can rebalance these disequilibria by removing VWF and heparanase-1 and by replacing protective ADAMTS13 and heparanase-2 [72]. Two meta-analysis even suggests a potential survival benefit triggering large mRCTs in both Europe (EXCHANGE-2, NCT05726825) and Canada (PLEXSIS, NCT05093075) that are about to start soon [73, 74] (Table 1).

## Immune augmentation strategies

### Immunostimulatory cytokines and growth factors

#### Granulocyte–macrophage colony-stimulating factor

Granulocyte–macrophage colony-stimulating factor (GM-CSF), a hematopoietic growth factor, restores HLA-DR expression on monocytes [75]. In sepsis patients with decreased monocytic HLA-DR, an mRCT demonstrated GM-CSF reduced the need for mechanical ventilation and increased TLR2/4-induced cytokines [76]. A meta-analysis found improved infection resolution, but no associated mortality benefit [77]. In a recent mRCT assessing HLA-DR-guided GM-CSF therapy's impact on ICU-acquired infection in immunosuppressed septic patients, no differences were observed in ICU-acquired infection or 28-day mortality [78]**.** The study ended prematurely after enrolling 98 of 166 planned patients, limiting conclusive findings. Comprehensive immunophenotyping beyond monocyte HLA-DR may be needed for better predictive enrichment. In light of this approach, a recent RCT in children with sepsis defined immunoparalysis treated with GM-CSF as an LPS-induced TNF production capacity < 200 pg/mL (NCT05266001) (Table 2).

**Table 2:** Recent clinical studies that target immunosuppression in sepsis*

Immune augmentation agent	Mechanism of action	Study population	Primary outcome	Results	Author, year of publication or NCT number
GM-CSF	Increased monocytic HLA-DR expression Increased monocytic production of TLR2/4-induced cytokines	*n* = 98, septic shock	Difference in the number of patients presenting ≥ 1 ICU-acquired infection at day 28 or ICU discharge	No significant difference; terminated due to insufficient recruitment	Vacheron et al. 2023 [78]
		*n* = 400, children with sepsis-induced multiple-organ dysfunction syndrome (MODS)	Cumulative 28-day Pediatric Logistic Organ Dysfunction (PELOD)-2 score	Recruiting	NCT05266001(GRACE2)
IFNγ	Primary activator of macrophages, neutrophils, and natural killer cells	*n* = 273, sepsis and septic shock	28-day mortality	No significant difference	Leventogiannis et al. 2022 [84]
		*n* = 280, sepsis (< 72 h)	Comparative decrease of the mean total Sequential Organ Failure Assessment (SOFA) score by at least 1.4 points by day 9 from randomization	Recruiting	Kotsaki et al. 2022 NCT04990232 (ImmunoSep) [85]
Thymosin alpha	Stimulates endogenous IFNy secretion and regulates T cell subsets	*n* = 1106, sepsis	28-day mortality	Pending	NCT02867267
		sepsis patients discharged after completing TESTS trial	3-year mortality	Pending	NCT04901104
Immunoglobulins (IgM-enriched)	Enhancement of pathogen recognition and clearance, scavenging of toxins and complement, neutralization of inflammatory cytokines	*n* = 200, sepsis or septic shock	Improvement of the mean multiple-organ failure score on day 7	Pending	NCT03334006
Mesenchymal stem cells (MSC)	Augmentation of bacterial clearance, limitation of apoptosis, injury repair enhancement	*n* = 9, septic shock	Safety, tolerability	Well tolerated, no infusion-associated, and serious adverse events	McIntyre et al. 2018 [101]
		*n* = 15, septic shock	Safety, tolerability	Well tolerated, no infusion-associated, and serious adverse events	He et al. 2018 [102]
		*n* = 114, sepsis and septic shock	Reduction in days on mechanical ventilation, or renal replacement therapy, or vasopressors; safety and tolerability	Pending	NCT03369275 (CISS2)
		*n* = 66, septic shock (≥ 2 organ failures other than hemodynamic)	Sofa score on day 7	Pending	NCT02883803 (CHOCMSC)
		*n* = 21, septic shock	Safety, tolerability	Pending	NCT04961658 (AMETHYST)
Immune checkpoint inhibitor nivolumab	Anti-programmed cell death (PD)-1 monoclonal antibody	*n* = 13, sepsis	Safety, tolerability, pharmakokinetics	Safe, well tolerated, increase in lymphocyte count and mHLA-DR expression	Watanabe et al. 2020 [114]
		*n* = 31, sepsis diagnosed ≥ 24 h before treatment, ≥ 1 organ dysfunction, and absolute lymphocyte count ≤ 1.1 × 10^3^ cells/μL	Safety, tolerability and pharmacokinetics over 90d	Safe, no cytokine release syndrome	Hotchkiss et al. 2019 [115]

*A summary of clinical studies over last past 5 years

#### Interferon gamma

Interferon gamma (IFNγ) activates macrophages, NK cells, and neutrophils, bolstering immune responses against pathogens [79]. In septic patients, low IFNγ-secretion is linked to secondary infection or death, while IFNγ-treatment increases HLA-DR expression and production of pro-inflammatory cytokines [80–83]. An mRCT on IFNγ for sepsis-related immune paralysis ended early due to slow enrollment (< 30% CD14 monocytes with HLA-DR) [84]. Another mRCT uses a cutoff of < 5000 HLA-DR receptors per CD14 monocyte for septic immunosuppression treatment [85] (Table 2). However, high IFNγ levels in early sepsis are associated with secondary candida infection, suggesting its role as an immunosuppressive mediator [86]. These negative effects might contribute to the early termination of another RCT to prevent hospital-acquired pneumonia in a heterogeneous group of patients, highlighting the need to stratify septic patients for IFNγ use in an immunosuppressive subphenotype [87].

#### Thymosin alpha 1

Thymosin alpha 1 (TA1) is a peptide synthesized primarily in the thymus gland and has long been known to modulate, enhance, and restore immune function. TA1 activates TLR2 and -9 in myeloid and dendritic cells, promoting adaptive responses and CD4+/CD8+ T cell maturation [88]. An mRCT with 367 septic patients showed increased monocyte HLA-DR expression and a trend toward improved survival (*p* = 0.06) in patients receiving TA1 [89]. A meta-analysis of 12 trials revealed lower sepsis mortality, but caution is needed due to individual study quality and size [90]. A large mRCT involving 1106 patients was recently completed. Its results may provide further insight into the therapeutic effect of TA1 in sepsis (NCT02867267 and NCT04901104, Table 2).

### Immunoglobulins

Intravenous immunoglobulins (IVIg) are used to neutralize microbes, reduce apoptosis of immune cells, limit inflammation, and mediate phagocytosis by macrophages. In septic patients, studies have demonstrated correlations between survival probability and concentrations of IgG, IgM, and IgA [91, 92]. RCTs on IVIg treatment in sepsis showed conflicting outcomes. Meta-analyses indicated reduced mortality with IVIg and IgM-enriched IVIg (IVIgM) [93, 94]. However, due to study quality variations, dosing differences, and control measures, evidence quality is low. Current guidelines advise against IVIg use in sepsis [34]. A large RCT is currently underway to investigate the effect of IVIgM therapy in sepsis patients (NCT03334006) (Table 2). This trial is monitoring several biomarkers, including Igs, cytokines, and cellular HLA-DR expression, to determine which subgroup of patients (those with hyperinflammation or immunosuppression) may benefit from IVIgM treatment.

### Mesenchymal stem cells

Multipotent mesenchymal stem cells (MSCs) hold promise for sepsis immunotherapy due to their immunomodulatory, antimicrobial, regenerative, and anti-apoptotic properties. In preclinical models, MSC application rebalances inflammation by suppressing pro-inflammatory cytokines and enhancing anti-inflammatory mediators [95, 96]. MSCs restore organ structure and function, including kidneys and liver, and boost phagocytic activity of monocytes against gram-negative sepsis [97, 98]. Meta-analyses indicate lower sepsis mortality with MSC therapy in animal models [99, 100]. Phase I trials on septic shock patients and COVID-19 ARDS cases showed MSCs' safety and limited adverse events [101–103]. Several ongoing phase I and II sepsis trials (NCT03369275, NCT02883803, NCT04961658) will provide more insights into MSC therapy's safety and efficacy (Table 2).

### Immune checkpoint inhibitors

Immune checkpoint receptors are important immune modulators that are critical for self-tolerance and regulation of ongoing immune responses. Several checkpoint receptors including programmed cell death protein 1 (PD-1), B and T lymphocyte attenuator (BTLA), and lymphocyte activation gene 3 (LAG-3), along with their respective ligands such as PD-L1, are upregulated on leukocytes during sepsis [104]. PD-1/PD-L1 upregulation on CD4+ lymphocytes and plasmacytoid dendritic cells is seen in sepsis-related immunosuppression [105]. Increased BTLA and PD-1 expression on CD4+ lymphocytes links to secondary infections, prolonged ICU stays, and higher mortality [106, 107]. Targeting PD-1/PD-L1 in preclinical studies counters apoptosis, restores cell function, and improves survival [108–110]. Ex vivo inhibition of the PD-1/PD-L1 pathway reduced apoptosis, improved immune cell function, and increased cytokine production in leukocytes from septic patients [111, 112]. In a case study of an immunocompromised patient with refractory fungal sepsis, it was observed that combined administration of anti-PD-1 antibody and IFNγ resulted in an increase in lymphocyte count and enhanced expression of monocytic HLA-DR [113]. The PD-1 inhibitor nivolumab was shown to be well tolerated in two-phase I trials conducted in immunocompromised patients with sepsis [114, 115] (Table 2). In these two trials, nivolumab also appeared to improve immune function by increasing monocytic HLA-DR expression and lymphocyte counts.

## Personalized immunotherapy

**Fig. 3:**
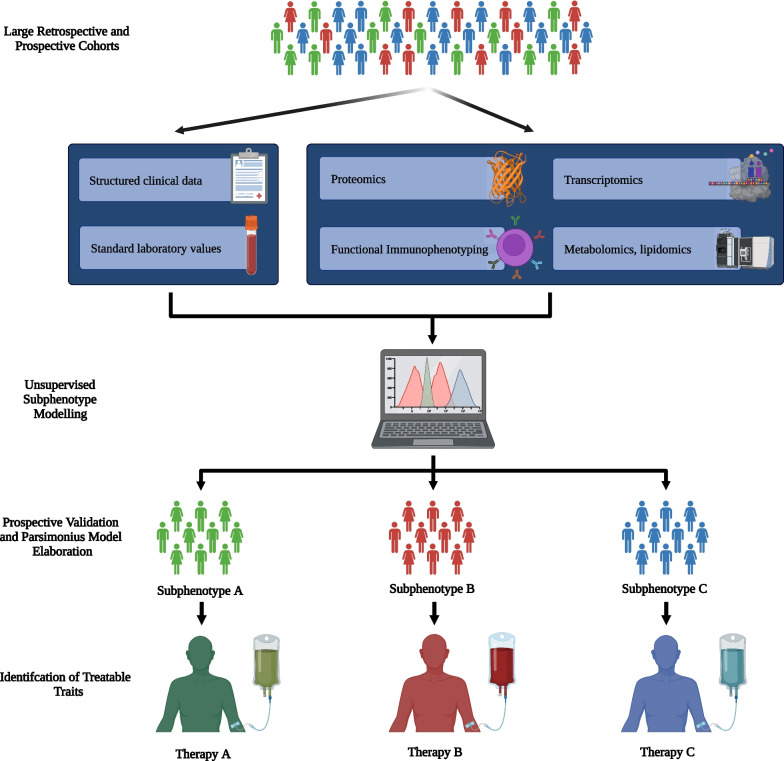
Overview of the potential research pathway leading from data to the identification of functional endotypes. First, clinical and biological data have to be collected in the framework of observational cohorts or randomized controlled trials. Critical relevance lies in the collection of samples that allow the implementation of high-throughput biological analyses in a second step. Optimally data from multiple databases are bundled in order to allow a robust subphenotype discovery. In a third step, data are fed into an unsupervised machine learning pipeline, which hopefully identifies clusters of patients in the given multi-dimensional variable constellation. These clusters or subphenotypes have then to be validated in an external prospective cohort, and optimally, a parsimonious model is then elaborated that allows identification of subphenotypes at the bedside with a minimal number of variables. Finally, and as the ultimate goal of phenotyping, a biological correlate or ideally, a treatable trait, is identified for each subphenotype, which can be targeted by means of a specific medication, leading to the transition from a subphenotype to a functional endotype

The heterogeneity of sepsis, spanning from its broad definition to the conundrum surrounding its pathophysiological inception and development, has hindered successful immunomodulatory therapies. Traditional subgrouping based on single traits or biomarkers falls short. Advances in computing and data have enabled investigating this diversity to find patient subgroups (subphenotypes) with shared characteristics, biological mechanisms, and treatment responses (Fig. 3). Modern subphenotyping relies on unsupervised clustering algorithms such as *k*-means clustering or the very prominently used latent class analysis. Briefly, these algorithms identify data clusters in multi-dimensional space to infer different subphenotypes based on these clusters, but can be influenced by cohort biases and data collection. The sepsis subphenotypes identified to date can be subdivided into two main groups:

### Clinical subphenotypes

Several studies have undergone efforts to identify clinical subendotypes. An example is Seymour and colleagues seminal work, identifying *α*, *β*, *γ*, and *δ* phenotypes via k-means clustering [116]. *α* had least organ dysfunction; *β* was older with comorbidities; *γ* and *δ* showed inflammation, with *δ* having higher lactate and vasoplegia. Mortality ranged from 2% (*α*) to 32% (*δ*), affecting intervention outcomes due to varied subphenotypes in trials. Similarly, Kudo et al. described four coagulopathy-based sepsis phenotypes, responding differently to recombinant human thrombomodulin [117]. Other studies investigated subphenotype-specific treatment responses in fluid resuscitation, antibiotic delay, temperature trajectories, progression to septic shock, and hemodynamics [118–122].

### Biological subphenotypes

This field covers protein-based biomarkers, proteomics, immune-phenotyping, transcriptomics, and metabolomics. Three subphenotypes based on whole-blood RNA patterns showed variations in glucocorticoid signaling, immunity, and zinc balance, linked to disease severity and mortality [123]. A multiplex messenger RNA quantification platform was developed, revealing distinct responses to glucocorticoid therapy [124, 125]. Davenport et al. identified two sepsis response signatures (SRS) from blood leucocyte transcriptomic clustering [126]. SRS1 subphenotype linked to higher mortality, while SRS2 showed endotoxin tolerance and T cell exhaustion, associating with HLA class II downregulation. In VANISH trial analysis, SRS2 had increased mortality with hydrocortisone therapy [127]. Other subphenotypes include molecular diagnosis and risk stratification for sepsis (MARS) subphenotypes from genome-wide mRNA clustering and “inflammopathic,” “adaptive,” and “coagulopathic subphenotypes from pooled transcriptomic data [128, 129]. Alternative sepsis subphenotyping methods involve flow cytometry-based immunophenotyping and combined transcriptomic, proteomic, and metabolomic data [130, 131]. The PROVIDE trial recently investigated whether a hyperinflammatory subphenotype (identified by serum ferritin > 4420 ng/mL) benefits from anakinra treatment and immunoparalyzed individuals (identified by < 5000 HLA-DR/monocytes) benefit from rhIFNy administration [84]. However, the 36 hyperinflammatory patients randomized to receive IL-1Ra or placebo before premature discontinuation of the study showed no difference in 28-day mortality. A follow-up study called the ImmunoSep trial with an expanded therapeutic window for the treatment of hyperinflammation by anakinra is currently recruiting [85].

## Summary and future perspectives

The sepsis syndrome arises from a complex dysregulation in the host's response to pathogens (Fig. 1). Immunotherapeutics demonstrated promising preclinical efficacy, yet clinical applicability struggles due a lack of in depth knowledge and efficient monitoring tools to individualize specific targeted treatment strategies [6]. Recognizing sepsis's heterogeneity as a key factor, initial distinct response phenotypes have been identified using both biomarkers and clinical data (Fig. 2) [116] showing their potential in post hoc subanalyses of prior negative RCTs pinpointing certain phenotypes potentially benefiting from immunotherapy [19, 116, 127]. The first prospective RCTs using biomarkers for such predictive enrichment are on the way [84, 85]. Granularity might be further raised by zooming-in on the “-omics” level describing so-called sepsis endotypes. Obviously complicated by practical implementation issues where new biomarkers that can be used in the ICU would be highly desirable (*ideally* by Point-of-Care devices). We have no doubts that identification of treatable traits through “-omics” technologies will improve our chances of a successful therapeutic immune modulation. Computational tools like artificial intelligence and machine learning approaches will leverage extensive clinical and immune data helping us to uncover such new treatable traits [132].

The role of the microbiome and host metabolism in shaping the response to infection is poorly understood. Understanding patients' immunologic and metabolic status pre-sepsis can reveal risk factors and immune balance targets. Sepsis is highly dynamic, and tracking immune changes remains challenging for tailored treatment. Longitudinal immune parameter recording, including biomarkers and cell responsiveness, will aid flexible treatment paths guiding the immune system toward optimal state [133].

It is important to acknowledge that many therapeutic concepts oversimplify sepsis by focusing solely on systemic inflammation somewhat neglecting that organ dysfunction is the common denominator that determines the transition from an uncomplicated infection to sepsis. These failing organs become dysfunctional [134] as a consequence of an insufficient tissue damage control response and mismatch of energy demands and supplies [135, 136]. Mechanisms behind disease progression to sepsis are unclear, but protective cellular responses to stress signals, called disease tolerance to infection, reduce infection-associated consequences [137]. Serum metabolome and proteome integration in humans supports the hypothesis of a dysregulated metabolism [138]. It is of surprise that little work has been done to directly target the organ dysfunction apart from the (upstream) immune response. A first clinical phase II study that investigates repurposing epirubicin to improve tissue damage control is currently recruiting patients (EPOS-1; NCT05033808) [139]). Further potential molecular metabolic targets such as lactate, glutamine, pyruvate, or ketone bodies have been identified in translational studies, but remain to be tested in a personalized manner in clinical trials [140–143].

In our opinion, the current separation between hyperinflammation and immunosuppression is not sufficient to stratify all septic patients for immunomodulatory approaches. We need to find better ways to investigate their host responses that are physiologically not separated but rather closely linked to one another. In addition to that, we need to seek a better understanding of organ dysfunction in the large group of patients without these extreme immune-dysregulations [84, 144]. Recent advances to personalize and monitor therapies should allow us to modulate immunity and improve disease tolerance of the individual septic patient.

## Data Availability

Not applicable.

## Abbreviations

ADAMTS13: A disintegrin and metalloproteinase with a thrombospondin type 1 motif, member 13
APC: Activated protein C
ARDS: Acute respiratory distress syndrome
ART-123: Recombinant thrombomodulin
bioADM: Bioactive adrenomedullin
BLTA: B and T lymphocyte attenuator
COVID-19: Coronavirus disease 2019
DAMPs: Damage-associated molecular patterns
delta-SOFA score: Delta-sequential organ failure assessment score
DIC: Disseminated intravascular coagulation
EC: Endothelial cells
eGC: Endothelial glycocalyx
eNOS: Endothelial nitric oxide synthase
HLA-DR: Human leukocyte antigen-DR isotype
ICU: Intensive care unit
iNOS: Inducible nitric oxide synthase
IFNy: Interferon gamma
IvIG: Intravenous immunoglobulins
LAG-3: Lymphocyte-activation gene 3
MARS: Molecular diagnosis and risk stratification for sepsis
mRCT: Multi-regional randomized controlled trials
MSC: Mesenchymal stem cell
PD-1: Programmed cell death protein 1
PD-L1: Programmed cell death ligand 1
RCT: Randomized controlled trial
SoC: Standard of care
SRS: Sepsis response signature
suPAR: Soluble urokinase-type plasminogen activator receptor
TA1: Thymosin alpha 1
TPE: Therapeutic plasma exchange
TTP: Thrombotic thrombocytopenic purpura
VSMC: Vascular smooth muscle cells
VWF: Von Willebrand factor

## References

[1] Rudd KE, Johnson SC, Agesa KM, Shackelford KA, Tsoi D, Kievlan DR (2020). Global, regional, and national sepsis incidence and mortality, 1990–2017: analysis for the global burden of disease study. The Lancet.

[2] van der Poll T, Shankar-Hari M, Wiersinga WJ (2021). The immunology of sepsis. Immunity.

[3] Wiersinga WJ, van der Poll T (2022). Immunopathophysiology of human sepsis. EBioMedicine.

[4] Singer M, Deutschman CS, Seymour CW, Shankar-Hari M, Annane D, Bauer M (2016). The third international consensus definitions for sepsis and septic shock (sepsis-3). JAMA.

[5] Lesch S, Gill S (2021). The promise and perils of immunotherapy. Blood Adv.

[6] Steinhagen F, Schmidt SV, Schewe JC, Peukert K, Klinman DM, Bode C (2020). Immunotherapy in sepsis—brake or accelerate?. Pharmacol Ther.

[7] Death by TNF: a road to inflammation: PubMed [Internet]. [cited 2023 Nov 12]. https://pubmed.ncbi.nlm.nih.gov/36380021/.

[8] Abraham E, Wunderink R, Silverman H, Perl TM, Nasraway S, Levy H (1995). Efficacy and safety of monoclonal antibody to human tumor necrosis factor alpha in patients with sepsis syndrome. A randomized, controlled, double-blind, multicenter clinical trial. TNF-alpha MAb Sepsis Study Group. JAMA.

[9] Abraham E, Glauser MP, Butler T, Garbino J, Gelmont D, Laterre PF (1997). p55 Tumor necrosis factor receptor fusion protein in the treatment of patients with severe sepsis and septic shock. A randomized controlled multicenter trial. Ro 45-2081 Study Group. JAMA.

[10] Abraham E, Anzueto A, Gutierrez G, Tessler S, San Pedro G, Wunderink R (1998). Double-blind randomised controlled trial of monoclonal antibody to human tumour necrosis factor in treatment of septic shock. NORASEPT II Study Group. Lancet.

[11] Cohen J, Carlet J (1996). INTERSEPT: an international, multicenter, placebo-controlled trial of monoclonal antibody to human tumor necrosis factor-alpha in patients with sepsis. International Sepsis Trial Study Group. Crit Care Med.

[12] Fisher CJ, Agosti JM, Opal SM, Lowry SF, Balk RA, Sadoff JC (1996). Treatment of septic shock with the tumor necrosis factor receptor: Fc fusion protein. The Soluble TNF Receptor Sepsis Study Group. N Engl J Med.

[13] Lv S, Han M, Yi R, Kwon S, Dai C, Wang R (2014). Anti-TNF-α therapy for patients with sepsis: a systematic meta-analysis. Int J Clin Pract.

[14] Panacek EA, Marshall JC, Albertson TE, Johnson DH, Johnson S, MacArthur RD (2004). Efficacy and safety of the monoclonal anti-tumor necrosis factor antibody F(ab’)2 fragment afelimomab in patients with severe sepsis and elevated interleukin-6 levels. Crit Care Med.

[15] Dinarello CA (2018). Overview of the IL-1 family in innate inflammation and acquired immunity. Immunol Rev.

[16] Opal SM, Fisher CJ, Dhainaut JF, Vincent JL, Brase R, Lowry SF (1997). Confirmatory interleukin-1 receptor antagonist trial in severe sepsis: a phase III, randomized, double-blind, placebo-controlled, multicenter trial. The Interleukin-1 Receptor Antagonist Sepsis Investigator Group. Crit Care Med.

[17] Fisher CJ, Dhainaut JF, Opal SM, Pribble JP, Balk RA, Slotman GJ (1994). Recombinant human interleukin 1 receptor antagonist in the treatment of patients with sepsis syndrome. Results from a randomized, double-blind, placebo-controlled trial. Phase III rhIL-1ra Sepsis Syndrome Study Group. JAMA.

[18] Meyer NJ, Reilly JP, Anderson BJ, Palakshappa JA, Jones TK, Dunn TG (2018). Mortality benefit of recombinant human interleukin-1 receptor antagonist for sepsis varies by initial interleukin-1 receptor antagonist plasma concentration. Crit Care Med.

[19] Shakoory B, Carcillo JA, Chatham WW, Amdur RL, Zhao H, Dinarello CA (2016). Interleukin-1 receptor blockade is associated with reduced mortality in sepsis patients with features of macrophage activation syndrome: reanalysis of a prior phase III trial. Crit Care Med.

[20] Kyriazopoulou E, Poulakou G, Milionis H, Metallidis S, Adamis G, Tsiakos K (2021). Early treatment of COVID-19 with anakinra guided by soluble urokinase plasminogen receptor plasma levels: a double-blind, randomized controlled phase 3 trial. Nat Med.

[21] de Nooijer AH, Pickkers P, Netea MG, Kox M (2023). Inflammatory biomarkers to predict the prognosis of acute bacterial and viral infections. J Crit Care.

[22] Le RQ, Li L, Yuan W, Shord SS, Nie L, Habtemariam BA (2018). FDA approval summary: tocilizumab for treatment of chimeric antigen receptor T cell-induced severe or life-threatening cytokine release syndrome. Oncologist.

[23] Shankar-Hari M, Vale CL, Godolphin PJ, Fisher D, Higgins JPT, WHO Rapid Evidence Appraisal for COVID-19 Therapies (REACT) Working Group (2021). Association between administration of IL-6 antagonists and mortality among patients hospitalized for COVID-19: a meta-analysis. JAMA.

[24] Ghosn L, Assi R, Evrenoglou T, Buckley BS, Henschke N, Probyn K (2023). Interleukin-6 blocking agents for treating COVID-19: a living systematic review. Cochrane Database Syst Rev.

[25] Hamilton FW, Thomas M, Arnold D, Palmer T, Moran E, Mentzer AJ (2023). Therapeutic potential of IL6R blockade for the treatment of sepsis and sepsis-related death: a Mendelian randomisation study. PLoS Med.

[26] Merle NS, Church SE, Fremeaux-Bacchi V, Roumenina LT (2015). Complement system part I: molecular mechanisms of activation and regulation. Front Immunol.

[27] Keshari RS, Popescu NI, Silasi R, Regmi G, Lupu C, Simmons JH (2021). Complement C5 inhibition protects against hemolytic anemia and acute kidney injury in anthrax peptidoglycan-induced sepsis in baboons. Proc Natl Acad Sci U S A.

[28] Silasi-Mansat R, Zhu H, Popescu NI, Peer G, Sfyroera G, Magotti P (2010). Complement inhibition decreases the procoagulant response and confers organ protection in a baboon model of *Escherichia coli* sepsis. Blood.

[29] Keshari RS, Silasi R, Popescu NI, Patel MM, Chaaban H, Lupu C (2017). Inhibition of complement C5 protects against organ failure and reduces mortality in a baboon model of *Escherichia coli* sepsis. Proc Natl Acad Sci U S A.

[30] Skjeflo EW, Sagatun C, Dybwik K, Aam S, Urving SH, Nunn MA (2015). Combined inhibition of complement and CD14 improved outcome in porcine polymicrobial sepsis. Crit Care.

[31] Bauer M, Weyland A, Marx G, Bloos F, Weber S, Weiler N (2021). Efficacy and safety of vilobelimab (IFX-1), a novel monoclonal anti-C5a antibody, in patients with early severe sepsis or septic shock-A randomized, placebo-controlled, double-blind, multicenter, phase IIA trial (SCIENS study). Crit Care Explor.

[32] Heming N, Sivanandamoorthy S, Meng P, Bounab R, Annane D (2018). Immune effects of corticosteroids in sepsis. Front Immunol.

[33] Pourmand A, Whiteside T, Yamane D, Rashed A, Mazer-Amirshahi M (2019). The controversial role of corticosteroids in septic shock. Am J Emerg Med.

[34] Evans L, Rhodes A, Alhazzani W, Antonelli M, Coopersmith CM, French C (2021). Surviving sepsis campaign: international guidelines for management of sepsis and septic shock 2021. Intensive Care Med.

[35] Horby P, Lim WS, Emberson JR, Mafham M, Bell JL, RECOVERY Collaborative Group (2021). Dexamethasone in hospitalized patients with Covid-19. N Engl J Med.

[36] Angus DC, Derde L, Al-Beidh F, Annane D, Arabi Y, Beane A (2020). Effect of hydrocortisone on mortality and organ support in patients with severe COVID-19: the REMAP-CAP COVID-19 corticosteroid domain randomized clinical trial. JAMA.

[37] Winkler MS, Osuchowski MF, Payen D, Torres A, Dickel S, Skirecki T (2022). Renaissance of glucocorticoids in critical care in the era of COVID-19: ten urging questions. Crit Care.

[38] Wu JY, Tsai YW, Hsu WH, Liu TH, Huang PY, Chuang MH (2023). Efficacy and safety of adjunctive corticosteroids in the treatment of severe community-acquired pneumonia: a systematic review and meta-analysis of randomized controlled trials. Crit Care.

[39] Dequin PF, Meziani F, Quenot JP, Kamel T, Ricard JD, Badie J (2023). Hydrocortisone in severe community-acquired pneumonia. N Engl J Med.

[40] Fowler AA, Truwit JD, Hite RD, Morris PE, DeWilde C, Priday A (2019). Effect of vitamin C infusion on organ failure and biomarkers of inflammation and vascular injury in patients with sepsis and severe acute respiratory failure: the CITRIS-ALI randomized clinical trial. JAMA.

[41] Fujii T, Luethi N, Young PJ, Frei DR, Eastwood GM, French CJ (2020). Effect of vitamin C, hydrocortisone, and thiamine vs hydrocortisone alone on time alive and free of vasopressor support among patients with septic shock: the VITAMINS randomized clinical trial. JAMA.

[42] Lamontagne F, Masse MH, Menard J, Sprague S, Pinto R, Heyland DK (2022). Intravenous vitamin C in adults with sepsis in the intensive care unit. N Engl J Med.

[43] Liang B, Su J, Shao H, Chen H, Xie B (2023). The outcome of IV vitamin C therapy in patients with sepsis or septic shock: a meta-analysis of randomized controlled trials. Crit Care.

[44] Sato R, Hasegawa D, Prasitlumkum N, Ueoka M, Nishida K, Takahashi K (2021). Effect of IV high-dose vitamin C on mortality in patients with sepsis: a systematic review and meta-analysis of randomized controlled trials. Crit Care Med.

[45] Sauer A, Putensen C, Bode C (2022). Immunomodulation by tetracyclines in the critically ill: an emerging treatment option?. Crit Care.

[46] Peukert K, Fox M, Schulz S, Feuerborn C, Frede S, Putensen C (2021). Inhibition of caspase-1 with tetracycline ameliorates acute lung injury. Am J Respir Crit Care Med.

[47] Bode C, Diedrich B, Muenster S, Hentschel V, Weisheit C, Rommelsheim K (2014). Antibiotics regulate the immune response in both presence and absence of lipopolysaccharide through modulation of Toll-like receptors, cytokine production and phagocytosis in vitro. Int Immunopharmacol.

[48] Colaço HG, Barros A, Neves-Costa A, Seixas E, Pedroso D, Velho T (2021). Tetracycline antibiotics induce host-dependent disease tolerance to infection. Immunity.

[49] Fredeking TM, Zavala-Castro JE, González-Martínez P, Moguel-Rodríguez W, Sanchez EC, Foster MJ (2015). Dengue patients treated with doxycycline showed lower mortality associated to a reduction in IL-6 and TNF levels. Recent Pat Anti-Infect Drug Discov.

[50] Sauer A, Peukert K, Putensen C, Bode C (2021). Antibiotics as immunomodulators: a potential pharmacologic approach for ARDS treatment. Eur Respir Rev.

[51] Karakike E, Scicluna BP, Roumpoutsou M, Mitrou I, Karampela N, Karageorgos A (2022). Effect of intravenous clarithromycin in patients with sepsis, respiratory and multiple organ dysfunction syndrome: a randomized clinical trial. Crit Care.

[52] Engelmann B, Massberg S (2013). Thrombosis as an intravascular effector of innate immunity. Nat Rev Immunol.

[53] Maneta E, Aivalioti E, Tual-Chalot S, Emini Veseli B, Gatsiou A, Stamatelopoulos K (2023). Endothelial dysfunction and immunothrombosis in sepsis. Front Immunol.

[54] Ranieri VM, Thompson BT, Barie PS, Dhainaut JF, Douglas IS, Finfer S (2012). Drotrecogin alfa (activated) in adults with septic shock. N Engl J Med.

[55] Lai PS, Thompson BT (2013). Why activated protein C was not successful in severe sepsis and septic shock: are we still tilting at windmills?. Curr Infect Dis Rep.

[56] Bernard GR, Vincent JL, Laterre PF, LaRosa SP, Dhainaut JF, Lopez-Rodriguez A (2001). Efficacy and safety of recombinant human activated protein C for severe sepsis. N Engl J Med.

[57] Ito T, Thachil J, Asakura H, Levy JH, Iba T (2019). Thrombomodulin in disseminated intravascular coagulation and other critical conditions-a multi-faceted anticoagulant protein with therapeutic potential. Crit Care.

[58] Vincent JL, Francois B, Zabolotskikh I, Daga MK, Lascarrou JB, Kirov MY (2019). Effect of a recombinant human soluble thrombomodulin on mortality in patients with sepsis-associated coagulopathy: the SCARLET randomized clinical trial. JAMA.

[59] Vincent JL, Ramesh MK, Ernest D, LaRosa SP, Pachl J, Aikawa N (2013). A randomized, double-blind, placebo-controlled, Phase 2b study to evaluate the safety and efficacy of recombinant human soluble thrombomodulin, ART-123, in patients with sepsis and suspected disseminated intravascular coagulation. Crit Care Med.

[60] Hagiwara A, Tanaka N, Uemura T, Matsuda W, Kimura A (2016). Can recombinant human thrombomodulin increase survival among patients with severe septic-induced disseminated intravascular coagulation: a single-centre, open-label, randomised controlled trial. BMJ Open.

[61] Valeriani E, Squizzato A, Gallo A, Porreca E, Vincent JL, Iba T (2020). Efficacy and safety of recombinant human soluble thrombomodulin in patients with sepsis-associated coagulopathy: a systematic review and meta-analysis. J Thromb Haemost.

[62] Aird WC (2003). The role of the endothelium in severe sepsis and multiple organ dysfunction syndrome. Blood.

[63] Laterre PF, Pickkers P, Marx G, Wittebole X, Meziani F, Dugernier T (2021). Safety and tolerability of non-neutralizing adrenomedullin antibody adrecizumab (HAM8101) in septic shock patients: the AdrenOSS-2 phase 2a biomarker-guided trial. Intensive Care Med.

[64] van Lier D, Picod A, Marx G, Laterre PF, Hartmann O, Knothe C (2022). Effects of enrichment strategies on outcome of adrecizumab treatment in septic shock: post-hoc analyses of the phase II adrenomedullin and outcome in septic shock 2 trial. Front Med (Lausanne).

[65] Wendel Garcia PD, Hilty MP, Held U, Kleinert EM, Maggiorini M (2021). Cytokine adsorption in severe, refractory septic shock. Intensive Care Med.

[66] Garbero E, Livigni S, Ferrari F, Finazzi S, Langer M, Malacarne P (2021). High dose coupled plasma filtration and adsorption in septic shock patients. Results of the COMPACT-2: a multicentre, adaptive, randomised clinical trial. Intensive Care Med.

[67] Becker S, Lang H, Vollmer Barbosa C, Tian Z, Melk A, Schmidt BMW (2023). Efficacy of CytoSorb®: a systematic review and meta-analysis. Crit Care.

[68] Stahl K, Wand P, Seeliger B, Wendel-Garcia PD, Schmidt JJ, Schmidt BMW (2022). Clinical and biochemical endpoints and predictors of response to plasma exchange in septic shock: results from a randomized controlled trial. Crit Care.

[69] Keith PD, Wells AH, Hodges J, Fast SH, Adams A, Scott LK (2020). The therapeutic efficacy of adjunct therapeutic plasma exchange for septic shock with multiple organ failure: a single-center experience. Crit Care.

[70] David S, Bode C, Putensen C, Welte T, Stahl K, EXCHANGE Study Group (2021). Adjuvant therapeutic plasma exchange in septic shock. Intensive Care Med.

[71] Pape T, Hunkemöller AM, Kümpers P, Haller H, David S, Stahl K (2021). Targeting the “sweet spot” in septic shock: a perspective on the endothelial glycocalyx regulating proteins Heparanase-1 and -2. Matrix Biol Plus.

[72] Stahl K, Hillebrand UC, Kiyan Y, Seeliger B, Schmidt JJ, Schenk H (2021). Effects of therapeutic plasma exchange on the endothelial glycocalyx in septic shock. Intensive Care Med Exp.

[73] Lee OPE, Kanesan N, Leow EH, Sultana R, Chor YK, Gan CS (2023). Survival benefits of therapeutic plasma exchange in severe sepsis and septic shock: a systematic review and meta-analysis. J Intensive Care Med.

[74] Rimmer E, Houston BL, Kumar A, Abou-Setta AM, Friesen C, Marshall JC (2014). The efficacy and safety of plasma exchange in patients with sepsis and septic shock: a systematic review and meta-analysis. Crit Care.

[75] Nierhaus A, Montag B, Timmler N, Frings DP, Gutensohn K, Jung R (2003). Reversal of immunoparalysis by recombinant human granulocyte-macrophage colony-stimulating factor in patients with severe sepsis. Intensive Care Med.

[76] Meisel C, Schefold JC, Pschowski R, Baumann T, Hetzger K, Gregor J (2009). Granulocyte-macrophage colony-stimulating factor to reverse sepsis-associated immunosuppression: a double-blind, randomized, placebo-controlled multicenter trial. Am J Respir Crit Care Med.

[77] Bo L, Wang F, Zhu J, Li J, Deng X (2011). Granulocyte-colony stimulating factor (G-CSF) and granulocyte-macrophage colony stimulating factor (GM-CSF) for sepsis: a meta-analysis. Crit Care.

[78] Vacheron CH, Lepape A, Venet F, Monneret G, Gueyffier F, Boutitie F (2023). Granulocyte-macrophage colony-stimulating factor (GM-CSF) in patients presenting sepsis-induced immunosuppression: the GRID randomized controlled trial. J Crit Care.

[79] Burke JD, Young HA (2019). IFN-γ: a cytokine at the right time, is in the right place. Semin Immunol.

[80] Döcke WD, Randow F, Syrbe U, Krausch D, Asadullah K, Reinke P (1997). Monocyte deactivation in septic patients: restoration by IFN-gamma treatment. Nat Med.

[81] Payen D, Faivre V, Miatello J, Leentjens J, Brumpt C, Tissières P (2019). Multicentric experience with interferon gamma therapy in sepsis induced immunosuppression. A case series. BMC Infect Dis.

[82] Grimm C, Dickel S, Grundmann J, Payen D, Schanz J, Zautner AE (2021). Case report: interferon-γ rescues monocytic human leukocyte antigen receptor (mHLA-DR) function in a COVID-19 patient with ARDS and superinfection with multiple MDR 4MRGN bacterial strains. Front Immunol.

[83] Delsing CE, Gresnigt MS, Leentjens J, Preijers F, Frager FA, Kox M (2014). Interferon-gamma as adjunctive immunotherapy for invasive fungal infections: a case series. BMC Infect Dis.

[84] Leventogiannis K, Kyriazopoulou E, Antonakos N, Kotsaki A, Tsangaris I, Markopoulou D (2022). Toward personalized immunotherapy in sepsis: the PROVIDE randomized clinical trial. Cell Rep Med.

[85] Kotsaki A, Pickkers P, Bauer M, Calandra T, Lupse M, Wiersinga WJ (2022). ImmunoSep (personalised immunotherapy in sepsis) international double-blind, double-dummy, placebo-controlled randomised clinical trial: study protocol. BMJ Open.

[86] Kim EY, Ner-Gaon H, Varon J, Cullen AM, Guo J, Choi J (2020). Post-sepsis immunosuppression depends on NKT cell regulation of mTOR/IFN-γ in NK cells. J Clin Invest.

[87] Roquilly A, Francois B, Huet O, Launey Y, Lasocki S, Weiss E (2023). Interferon gamma-1b for the prevention of hospital-acquired pneumonia in critically ill patients: a phase 2, placebo-controlled randomized clinical trial. Intensive Care Med.

[88] Dominari A, Hathaway Iii D, Pandav K, Matos W, Biswas S, Reddy G (2020). Thymosin alpha 1: a comprehensive review of the literature. World J Virol.

[89] Wu J, Zhou L, Liu J, Ma G, Kou Q, He Z (2013). The efficacy of thymosin alpha 1 for severe sepsis (ETASS): a multicenter, single-blind, randomized and controlled trial. Crit Care.

[90] Li C, Bo L, Liu Q, Jin F (2015). Thymosin alpha1 based immunomodulatory therapy for sepsis: a systematic review and meta-analysis. Int J Infect Dis.

[91] Tamayo E, Fernández A, Almansa R, Carrasco E, Goncalves L, Heredia M (2012). Beneficial role of endogenous immunoglobulin subclasses and isotypes in septic shock. J Crit Care.

[92] Bermejo-Martín JF, Rodriguez-Fernandez A, Herrán-Monge R, Andaluz-Ojeda D, Muriel-Bombín A, Merino P (2014). Immunoglobulins IgG1, IgM and IgA: a synergistic team influencing survival in sepsis. J Intern Med.

[93] Busani S, Damiani E, Cavazzuti I, Donati A, Girardis M (2016). Intravenous immunoglobulin in septic shock: review of the mechanisms of action and meta-analysis of the clinical effectiveness. Minerva Anestesiol.

[94] Cui J, Wei X, Lv H, Li Y, Li P, Chen Z (2019). The clinical efficacy of intravenous IgM-enriched immunoglobulin (pentaglobin) in sepsis or septic shock: a meta-analysis with trial sequential analysis. Ann Intensive Care.

[95] Gorman E, Millar J, McAuley D, O’Kane C (2021). Mesenchymal stromal cells for acute respiratory distress syndrome (ARDS), sepsis, and COVID-19 infection: optimizing the therapeutic potential. Expert Rev Respir Med.

[96] Schlosser K, Wang JP, Dos Santos C, Walley KR, Marshall J, Fergusson DA (2019). Effects of mesenchymal stem cell treatment on systemic cytokine levels in a phase 1 dose escalation safety trial of septic shock patients. Crit Care Med.

[97] Cóndor JM, Rodrigues CE, de Sousa Moreira R, Canale D, Volpini RA, Shimizu MHM (2016). Treatment with human Wharton’s jelly-derived mesenchymal stem cells attenuates sepsis-induced kidney injury, liver injury, and endothelial dysfunction. Stem Cells Transl Med.

[98] Krasnodembskaya A, Samarani G, Song Y, Zhuo H, Su X, Lee JW (2012). Human mesenchymal stem cells reduce mortality and bacteremia in gram-negative sepsis in mice in part by enhancing the phagocytic activity of blood monocytes. Am J Physiol Lung Cell Mol Physiol.

[99] Sun XY, Ding XF, Liang HY, Zhang XJ, Liu SH, Bing-Han (2020). Efficacy of mesenchymal stem cell therapy for sepsis: a meta-analysis of preclinical studies. Stem Cell Res Ther.

[100] Ge L, Zhao J, Deng H, Chen C, Hu Z, Zeng L (2021). Effect of bone marrow mesenchymal stromal cell therapies in rodent models of sepsis: a meta-analysis. Front Immunol.

[101] McIntyre LA, Stewart DJ, Mei SHJ, Courtman D, Watpool I, Granton J (2018). Cellular immunotherapy for septic shock. A phase I clinical trial. Am J Respir Crit Care Med.

[102] He X, Ai S, Guo W, Yang Y, Wang Z, Jiang D (2018). Umbilical cord-derived mesenchymal stem (stromal) cells for treatment of severe sepsis: aphase 1 clinical trial. Transl Res.

[103] Monsel A, Hauw-Berlemont C, Mebarki M, Heming N, Mayaux J, Nguekap Tchoumba O (2022). Treatment of COVID-19-associated ARDS with mesenchymal stromal cells: a multicenter randomized double-blind trial. Crit Care.

[104] McBride MA, Patil TK, Bohannon JK, Hernandez A, Sherwood ER, Patil NK (2020). Immune checkpoints: novel therapeutic targets to attenuate sepsis-induced immunosuppression. Front Immunol.

[105] Boomer JS, To K, Chang KC, Takasu O, Osborne DF, Walton AH (2011). Immunosuppression in patients who die of sepsis and multiple organ failure. JAMA.

[106] Shubin NJ, Monaghan SF, Heffernan DS, Chung CS, Ayala A (2013). B and T lymphocyte attenuator expression on CD4+ T-cells associates with sepsis and subsequent infections in ICU patients. Crit Care.

[107] Guignant C, Lepape A, Huang X, Kherouf H, Denis L, Poitevin F (2011). Programmed death-1 levels correlate with increased mortality, nosocomial infection and immune dysfunctions in septic shock patients. Crit Care.

[108] Zhang Y, Zhou Y, Lou J, Li J, Bo L, Zhu K (2010). PD-L1 blockade improves survival in experimental sepsis by inhibiting lymphocyte apoptosis and reversing monocyte dysfunction. Crit Care.

[109] Brahmamdam P, Inoue S, Unsinger J, Chang KC, McDunn JE, Hotchkiss RS (2010). Delayed administration of anti-PD-1 antibody reverses immune dysfunction and improves survival during sepsis. J Leukoc Biol.

[110] Phares TW, Kotraiah V, Chung CS, Unsinger J, Mazer M, Remy KE (2021). A peptide-based checkpoint immunomodulator alleviates immune dysfunction in murine polymicrobial sepsis. Shock.

[111] Chang K, Svabek C, Vazquez-Guillamet C, Sato B, Rasche D, Wilson S (2014). Targeting the programmed cell death 1: programmed cell death ligand 1 pathway reverses T cell exhaustion in patients with sepsis. Crit Care.

[112] Patera AC, Drewry AM, Chang K, Beiter ER, Osborne D, Hotchkiss RS (2016). Frontline science: defects in immune function in patients with sepsis are associated with PD-1 or PD-L1 expression and can be restored by antibodies targeting PD-1 or PD-L1. J Leukoc Biol.

[113] Grimaldi D, Pradier O, Hotchkiss RS, Vincent JL (2017). Nivolumab plus interferon-γ in the treatment of intractable mucormycosis. Lancet Infect Dis.

[114] Watanabe E, Nishida O, Kakihana Y, Odani M, Okamura T, Harada T (2020). Pharmacokinetics, pharmacodynamics, and safety of nivolumab in patients with sepsis-induced immunosuppression: a multicenter, open-label phase 1/2 study. Shock.

[115] Hotchkiss RS, Colston E, Yende S, Crouser ED, Martin GS, Albertson T (2019). Immune checkpoint inhibition in sepsis: a Phase 1b randomized study to evaluate the safety, tolerability, pharmacokinetics, and pharmacodynamics of nivolumab. Intensive Care Med.

[116] Seymour CW, Kennedy JN, Wang S, Chang CCH, Elliott CF, Xu Z (2019). Derivation, validation, and potential treatment implications of novel clinical phenotypes for sepsis. JAMA.

[117] Kudo D, Goto T, Uchimido R, Hayakawa M, Yamakawa K, Abe T (2021). Coagulation phenotypes in sepsis and effects of recombinant human thrombomodulin: an analysis of three multicentre observational studies. Crit Care.

[118] Zhang Z, Zhang G, Goyal H, Mo L, Hong Y (2018). Identification of subclasses of sepsis that showed different clinical outcomes and responses to amount of fluid resuscitation: a latent profile analysis. Crit Care.

[119] Han X, Spicer A, Carey KA, Gilbert ER, Laiteerapong N, Shah NS (2021). Identifying high-risk subphenotypes and associated harms from delayed antibiotic orders and delivery. Crit Care Med.

[120] Bhavani SV, Carey KA, Gilbert ER, Afshar M, Verhoef PA, Churpek MM (2019). Identifying novel sepsis subphenotypes using temperature trajectories. Am J Respir Crit Care Med.

[121] Liu R, Greenstein JL, Fackler JC, Bembea MM, Winslow RL (2020). Spectral clustering of risk score trajectories stratifies sepsis patients by clinical outcome and interventions received. Elife.

[122] Geri G, Vignon P, Aubry A, Fedou AL, Charron C, Silva S (2019). Cardiovascular clusters in septic shock combining clinical and echocardiographic parameters: a post hoc analysis. Intensive Care Med.

[123] Wong HR, Cvijanovich N, Lin R, Allen GL, Thomas NJ, Willson DF (2009). Identification of pediatric septic shock subclasses based on genome-wide expression profiling. BMC Med.

[124] Wong HR, Wheeler DS, Tegtmeyer K, Poynter SE, Kaplan JM, Chima RS (2010). Toward a clinically feasible gene expression-based subclassification strategy for septic shock: proof of concept. Crit Care Med.

[125] Wong HR, Cvijanovich NZ, Anas N, Allen GL, Thomas NJ, Bigham MT (2015). Developing a clinically feasible personalized medicine approach to pediatric septic shock. Am J Respir Crit Care Med.

[126] Davenport EE, Burnham KL, Radhakrishnan J, Humburg P, Hutton P, Mills TC (2016). Genomic landscape of the individual host response and outcomes in sepsis: a prospective cohort study. Lancet Respir Med.

[127] Antcliffe DB, Burnham KL, Al-Beidh F, Santhakumaran S, Brett SJ, Hinds CJ (2019). Transcriptomic signatures in sepsis and a differential response to steroids. From the VANISH randomized trial. Am J Respir Crit Care Med.

[128] Scicluna BP, van Vught LA, Zwinderman AH, Wiewel MA, Davenport EE, Burnham KL (2017). Classification of patients with sepsis according to blood genomic endotype: a prospective cohort study. Lancet Respir Med.

[129] Sweeney TE, Azad TD, Donato M, Haynes WA, Perumal TM, Henao R (2018). Unsupervised analysis of transcriptomics in bacterial sepsis across multiple datasets reveals three robust clusters. Crit Care Med.

[130] Leijte GP, Rimmelé T, Kox M, Bruse N, Monard C, Gossez M (2020). Monocytic HLA-DR expression kinetics in septic shock patients with different pathogens, sites of infection and adverse outcomes. Crit Care.

[131] Neyton LPA, Zheng X, Skouras C, Doeschl-Wilson A, Gutmann MU, Uings I (2022). Molecular patterns in acute pancreatitis reflect generalizable endotypes of the host response to systemic injury in humans. Ann Surg.

[132] Warnat-Herresthal S, Schultze H, Shastry KL, Manamohan S, Mukherjee S, Garg V (2021). Swarm learning for decentralized and confidential clinical machine learning. Nature.

[133] Schuurman AR, Sloot PMA, Wiersinga WJ, van der Poll T (2023). Embracing complexity in sepsis. Crit Care.

[134] Takasu O, Gaut JP, Watanabe E, To K, Fagley RE, Sato B (2013). Mechanisms of cardiac and renal dysfunction in patients dying of sepsis. Am J Respir Crit Care Med.

[135] Singer M (2008). Cellular dysfunction in sepsis. Clin Chest Med.

[136] Soares MP, Gozzelino R, Weis S (2014). Tissue damage control in disease tolerance. Trends Immunol.

[137] Medzhitov R, Schneider DS, Soares MP (2012). Disease tolerance as a defense strategy. Science.

[138] Langley RJ, Tsalik EL, van Velkinburgh JC, Glickman SW, Rice BJ, Wang C (2013). An integrated clinico-metabolomic model improves prediction of death in sepsis. Sci Transl Med.

[139] Figueiredo N, Chora A, Raquel H, Pejanovic N, Pereira P, Hartleben B (2013). Anthracyclines induce DNA damage response-mediated protection against severe sepsis. Immunity.

[140] Vandewalle J, Timmermans S, Paakinaho V, Vancraeynest L, Dewyse L, Vanderhaeghen T (2021). Combined glucocorticoid resistance and hyperlactatemia contributes to lethal shock in sepsis. Cell Metab.

[141] Leitner BP, Lee WD, Zhu W, Zhang X, Gaspar RC, Li Z (2023). Tissue-specific reprogramming of glutamine metabolism maintains tolerance to sepsis. PLoS ONE.

[142] McCall CE, Zhu X, Zabalawi M, Long D, Quinn MA, Yoza BK (2022). Sepsis, pyruvate, and mitochondria energy supply chain shortage. J Leukoc Biol.

[143] Karagiannis F, Peukert K, Surace L, Michla M, Nikolka F, Fox M (2022). Impaired ketogenesis ties metabolism to T cell dysfunction in COVID-19. Nature.

[144] Powell RE, Soares MP, Weis S. What’s new in intensive care: disease tolerance. Intensive Care Med. 2023;49(10):1235–1237. 10.1007/s00134-023-07130-8. 10.1007/s00134-023-07130-8 PMC10556172 37353606

